# Improving the Mechanical and Surface Properties of Aramid Fiber by Grafting with 1,4-Dichlorobutane under Supercritical Carbon Dioxide

**DOI:** 10.3390/ma12223766

**Published:** 2019-11-16

**Authors:** Chuyuan Jia, Chengce Yuan, Zhenyu Ma, Yunzhe Du, Li Liu, Yudong Huang

**Affiliations:** MIIT Key Laboratory of Critical Materials Technology for New Energy Conversion and Storage, School of Chemistry and Chemical Engineering, Harbin Institute of Technology, Harbin 150001, China16b925019@stu.hit.edu.cn (C.Y.); duyunzhehit@outlook.com (Y.D.); ydhuang.hit1@aliyun.com (Y.H.)

**Keywords:** aramid fiber, supercritical CO_2_, composites, surface modification, mechanical properties

## Abstract

The mechanical and surface properties of aramid fiber were simultaneously improved by grafting with 1,4-dichlorobutane in supercritical carbon dioxide (scCO_2_). 1,4-dichlorobutane was penetrated and reacted with heterocyclic an aromatic polyamide backbone along with supercritical CO_2_ fluids. The surface roughness and surface energy of the modified aramid fiber—which were measured by scanning electron microscopy (SEM) and the dynamic contact angle (DCA) test, respectively—significantly increased. X-ray diffractometer (XRD) measurements indicated that the crystallinity of the aramid fiber obviously increased after treatment in scCO_2_ under stretching. A single fiber tensile test showed that the tensile strength of the aramid fiber greatly enhanced after the modification due to its improved crystallinity characteristics. Moreover, the monofilament pull-out tests indicated that the interfacial shear strength (IFSS) test of the aramid fiber/epoxy composite increased by 24.3% from 51.30 to 63.91 MPa after the modification. This research provides a novel method for the simultaneous surface modification and mechanical improvement of aramid fiber properties.

## 1. Introduction

With the rapid development of the aerospace field, fiber-reinforced, resin-based composite materials have gradually replaced traditional metal materials due to their advantages of low density and high strength. Structural fibers such as carbon, glass and aramid fibers have been intensively adopted in high-performance applications. Among them, aramid fiber is unique for its inherent “skin-core” structure. The “skin” is constituted of highly crystalline rigid molecular chains, packed up regularly and tightly, arranged along the axial direction of the fiber. The “core” is composed of a large number of rod-shaped macromolecules which depend on hydrogen bond cross-linking. However, the poor compatibility between aramid fibers and resin matrixes, which is due to the inert and smooth surface of the aramid fiber, directly leads to the poor interfacial performance of the composites [1,2].

The high proportion of aromatic rings in the molecular segment leads to a large steric hindrance and a weak interaction between the molecular chains. Moreover, the inert surface of aramid fibers results in a weak bonding force between the fiber and the resin matrix due to the lack of active functional groups [3,4,5,6]. The unfavorable factors have restricted the mechanical performance of aramid fiber-reinforced resin composites and the further development of their application. Therefore, it is urgent and necessary to carry out modification research on aramid fibers, improving the interfacial performance between the fiber and the resin matrix, in order to attain a higher application efficiency of aramid fibers and the expansive application fields of aramid fibers. Researchers have already accomplished a lot of research on the modification of aramid fibers, and they have achieved some ideal results in the past few decades.

The surface modification technology of aramid fibers mainly includes physical modification technology [7,8,9,10,11], chemical modification technology [12,13], and other modification technologies, including the biological enzymatic method and rare earth modification. [14,15]. Remarkably, the treatment of fibers or synthetic polymeric materials with supercritical fluid (SCF) has become a hot research issue in recent years. The role of supercritical fluids in polymers mainly involves the dissolution, swelling, plasticization and promotion of crystallization [16,17,18]. The critical temperature of commonly-used supercritical fluids is higher than the heat-resistant temperature of most polymeric materials, which endows supercritical carbon dioxide (scCO_2_) a unique advantage in dissolving and swelling polymer materials to provide preconditions for further research. In recent years, a large number of literature has reported on the surface modification of aramid fibers by scCO_2_ to prepare functional aramid fibers. Qin et al. applied scCO_2_ to the treatment of aramid fibers and isocyanate-terminated, liquid nitrile rubber to improve the adhesion of vinyl epoxy composites. The flexural strength and interlaminar shear strength (ILSS) of the treated aramid fiber/vinyl epoxy composite increased by 18.1% and 28.9%, respectively [19]. Zheng et al. studied the effect of treatment pressure on the structure and properties of PMIA fibers. The results showed that PMIA surface morphology, water contact angle, macromolecular interaction, crystal structure, thermal properties and tensile strength changed during supercritical carbon dioxide treatment [20]. Kanbargi et al. [21] proposed a new method to pretreat fibers with a coupling agent in the presence of scCO_2_ to improve the adhesion between poly (p-phenylene terephthalamide) fibers and tire-reinforced natural rubber.

However, most of the current research has improved interfacial properties at the expense of tensile strength. In this paper, under the condition of static stretching, supercritical carbon dioxide that carrying 1,4-dichlorobutane molecules infiltrated the fibers to initiate a chemical reaction, both on the surface and the interior part of the aramid fibers. The optimized temperature, pressure and time of supercritical condition were investigated. Then, gradient experiments were carried out by changing the stretching conditions, and the effects of different stretching conditions on fiber crystallization and mechanical properties were discussed. After the modification, the surface properties such as the surface morphology, surface energy and reaction activity of the aramid fibers were improved significantly. The improved interfacial performance and tensile strength were achieved at the same time by using the modification method above. Finally, an enhanced interface shear strength (IFSS) of the aramid fibers/epoxy composite was achieved.

## 2. Materials and Methods

### 2.1. Materials

Aramid fibers with an average single filament diameter of 18.8 μm were provided by the China Aerospace Science and Technology Group 46th Research Institute (Huhhot, China). Carbon dioxide was purchased from Harbin LiMing Gas Co., Ltd. (Harbin, China). 1,4-dichlorobutane (98% pure) and octane (96% pure) were both purchased from Aladdin (Shanghai, China). E51 epoxy resin and acetone, provided, respectively, by Wuxi Resin Factory (Wuxi, China) and Jinfeng Chemical Co., Ltd. (Tianjin, China), were used as received.

### 2.2. Methods

#### 2.2.1. Desizing

The aramid fibers were extracted with acetone for 24 h in a Soxhlet extractor to remove the sizing agent, as well as the spinning and knitting waxes and oils. After extraction, the fibers were dried at 70 °C for 5 h in an air oven.

#### 2.2.2. Treatment in scCO_2_

The aramid fibers were placed in the high-pressure stainless steel vessel (1 L) where treatment was performed. The corresponding volume of the 1,4-dichlorobutane was added to the vessel, and the mass-to-volume ratio of the fiber to the reagent was 1:60. When the vessel reached the setting temperature, CO_2_ gas was introduced via a high-pressure syringe pump. After a certain period of time, the heating was stopped. Finally, when the vessel reached room temperature, the treated fibers were taken out of the vessel, washed with acetone in a Soxhlet extractor, and dried under the same conditions mentioned above. In the scCO_2_ treatment, pressure, time and temperature were controlled to obtain optimized modified parameters. For pressure, 7.5, 9 and 10 MPa were chosen as the conditions. For time, 40, 60 and 90 min were chosen as the conditions. For temperature, 40, 60 and 80 °C were chosen as the conditions. Seven samples were prepared according to the three conditions; the details are presented in Table 1.

Then the aramid fibers were fixed on a self-made device, and a series of tensile forces of 0, 3, 6, 9, 12, and 15 N were applied along the fibers. The stretched fibers were placed in the high-pressure stainless steel vessel and treated with scCO_2_.

#### 2.2.3. Characterization and Performance Study of Composites

ATR-IR spectra were measured on a Thermo Fisher Nicoleti S50 (Thermo Fisher Scientific, Waltham, MA, USA) in the range of 650–4000 cm^−1^ with a resolution of 0.4 cm^−1^. Surface morphologies of the fibers were observed using a ZEISS SUPRA 55 field emission scanning electron microscope (SEM, ZEISS, Oberkochen, Germany). X-ray photoelectron spectroscopy (XPS, ESCALAB 220i-XL, VG Scientific ltd., London, UK) was employed to analyze the surface chemical composition of the fiber. The Ar ion gun etch with XPS measurements was used to investigate the depth profile of the sample after treatment for 60 s. An X-ray diffractometer (XRD, RIGAKU D/MAX-rβ, Tokyo, Japan) was used to study the crystallite information of the fiber. Before the measurement, the modified aramid fiber was cleaned with acetone in the Soxhlet extractor for 24 h and dried in a vacuum oven to remove the residual chemical reagent.

A contact angle meter (DCAT21, Data-Physics Instrument, Filderstadt, Germany) was used to investigate the surface wettability of the aramid fibers. Deionized water (*γ* = 72.8 mJ/m^2^, *γ^d^* = 21.8 mJ/m^2^) and diiodomethane (*γ* = 50.8 mJ/m^2^, *γ^d^* = 50.8 mJ/m^2^) were used as the testing liquids. The dynamic contact angle was determined from the mass change during the immersion of the aramid fibers into testing liquids. The surface free energy (*γ*), dispersive component (*γ^d^*) and polar component (*γ^f^*) of the aramid fibers were estimated from the measured dynamic contact angle of the testing liquids with known surface tension components. All specimens were tested for 10 samples, and the average value was considered as the tested value. A single filament tensile test was applied to evaluate the mechanical property on a universal testing machine (Instron 5500R, Instron, Norwood, MA, USA) according to the ASTM D3379-75. At least 60 samples were tested at a cross-head speed of 1 mm/min. Fiber samples that broke at the gripped portion in the tensile test were removed.

The interfacial shear strength (IFSS) test was adopted to quantify the interfacial property between the aramid fibers and the resin matrix by the monofilament pull-out measurement (equipment for evaluation of the fiber/resin composite interface properties, Tohei Sangyo Co., Ltd., Tokyo, Japan). The E51 and H256 were mixed at the weight ratio of 100:32 to prepare micro-composites. The micro-composites were cured at 90 °C for 2 h, 120 °C for 2 h and 150 °C for 2 h. After curing, an epoxy droplet was blocked on the single fiber and loaded by two metal blades at a cross-head speed of 0.06 mm·min^−1^ during the test. The value of the IFSS was calculated according to Equation (1)

IFSS = F/(πDL)(1)

where F is the value of maximum load, D is the average diameter of the aramid fibers, and L is the embedded length of the fiber sample in the matrix. All specimens were tested for 20 samples, and the average value was considered as the tested value.

## 3. Results and Discussion

### 3.1. Effect of Different Treatment Conditions on Surface Characteristic of Aramid Fiber

The aramid fiber was soaked in scCO_2_ that contained 1,4-dichlorobutane. In the scCO_2_ treatment, the three conditions of pressure, time and temperature could be controlled to obtain the optimized modified parameters. As presented in Table 1, seven samples were prepared according to the three conditions.

**Figure 1:**
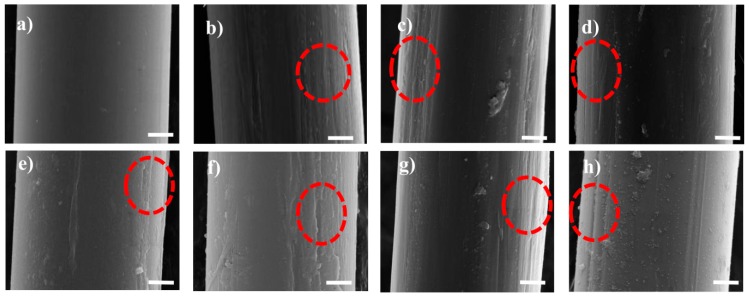
SEM images of aramid fiber surface morphology before and after scCO_2_ treatment. (**a**) untreated fiber; (**b**–**h**) modified fiber of samples 1–7 in Table 1; the scale bar is of 2 μm.

The SEM images indicated that the surface roughness of the aramid fiber obviously increased after the interaction of 1,4-dichlorobutane in the scCO_2_ environment, as shown in Figure 1. A large number of gullies and protrusions appeared on the surface of the treated aramid fiber. Obvious undulation suggested the successful interaction between fibers and 1,4-dichlorobutan in the supercritical environment. The chlorine-containing compound was deposited on the fiber surface. However, the surface morphologies of the aramid fibers treated with pure scCO_2_ did not show obvious changes as presented in Figure S1.

**Figure 2:**
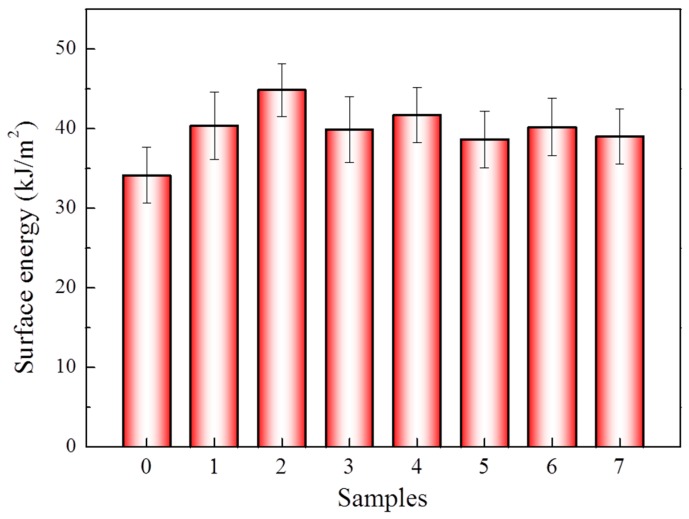
Surface energy of the aramid fibers before and after being treated with different conditions. (0): untreated fiber; (1–7): modified fiber of samples 1–7 in Table 1. All specimens were tested for 10 samples, and the average value was considered to be the tested value. The vertical bars represent the standard deviations in the measurement.

Moreover, the surface energies of the aramid fiber of different samples were measured. As presented in Figure 2, the surface energy of the aramid fibers was improved after the modification. For sample 2, the surface energy increased from 34.15 to 44.87 kJ/m^2^ with an increment of 31.39%. The improvement of surface energy could be attributed to the fiber surface roughness and the increase of the surface polar functional groups of the fiber. The aramid fiber with a higher surface energy presented a better compatibility of epoxy resin and could thus have the stronger interface property of the composites.

**Figure 3:**
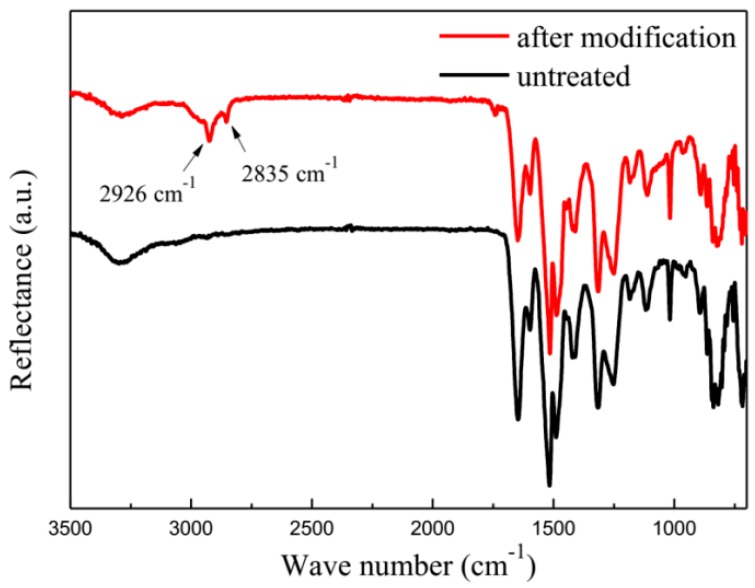
FTIR spectra of the aramid fiber before and after the modification of sample 2 in Table 1.

To investigate the mechanism of the improved surface property, FTIR was used to verify the interaction between the fibers and 1,4-dichlorobutane. As shown in Figure 3, it can be clearly seen that obvious absorption peak at 2853 cm^−1^ appeared in the modified fiber compared with the 2926 cm^−1^ peak of the untreated aramid fiber, which is related to the symmetrical stretching vibrational of CH_2_. This indicated that 1,4-dichlorobutane successfully grafted onto the surface of the fiber. The 1,4-dichlorobutane with the polar group could significantly improve the surface energy of the aramid fibers and also enhance the surface activity and roughness of the aramid fibers.

**Figure 4:**
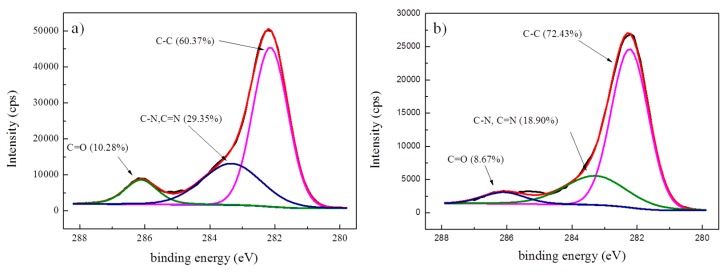
C1s spectra and concentrations of the correlative functional groups of the aramid fiber samples. (**a**) Untreated fibers and (**b**) modified fibers of sample 2 in Table 1.

In addition, the XPS spectra of the aramid fiber were measured before and after modification in scCO_2_ with 1,4-dichlorobutane. The Ar ion gun etch with XPS measurements was also used to investigate the depth profile of the sample after treatment. As presented Figure 4 and Figure S2, it can be clearly seen that, compared with the untreated fibers, the relative content of C–C increased significantly at the surface and inside. Thus 1,4-dichlorobutane was successfully grafted on the aramid fiber.

According to the deconvolution of the C1s peaks, the functional group contents were calculated, as listed in Table 2. On the surface, the relative content of C–C increased from 60.37% to 72.43%, and the contents of the C–N, C=N and C=O groups decreased because a layer of the 1,4-dichlorobutane molecule was absorbed on the aramid fiber surface. The relative content of C–C inside the modified fiber was 64.72%, which obviously decreased compared to the modified fiber surface, and the contents of C–N, C=N and C=O group relatively increased.

Moreover, the element contents were measured by using XPS. As presented in Table 3, the C/O atomic ratio obviously increased after modification by scCO_2_ that contained 1,4-dichlorobutane. Relatively, the Cl content of the aramid fibers significantly increased after treatment. The reaction between 1,4-dichlorobutane and the macromolecule chain could well-explain the increase, which was also testified by the change of the C/O atomic ratios and the element composition of Cl, as shown in Table 3.

The C/O atomic ratio of the untreated fiber was 4.80%, and the ratio of the fiber after treatment was 28.81%. The element compositions of Cl shared a similar changing tendency, from 0.04% to 1.93%. Among them, the C/O atomic ratio and the chlorine composition of the fiber surface were higher than the untreated one, which was due to the fact that 1,4-dichlorobutane reacted with the aramid fibers. In addition, compared with the modified fiber surface, both the C/O atomic ratio and the element composition of Cl slightly decreased after the ion etching of the fiber.

### 3.2. Effect of Different Stretching Conditions on Mechanical Properties of Aramid Fibers

**Figure 5:**
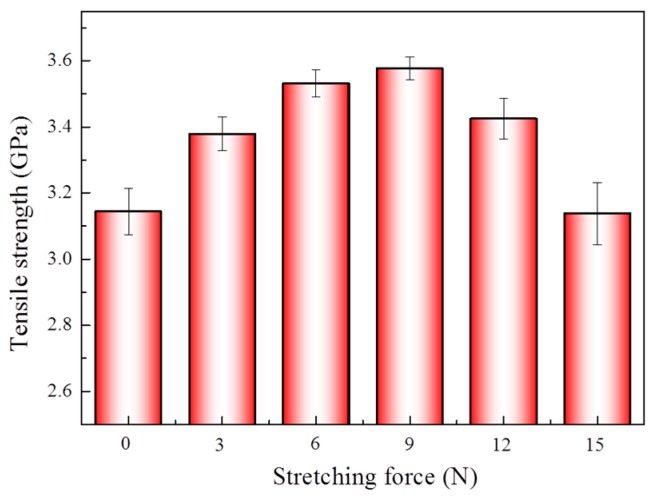
Tensile strength of the fiber under different tensile forces in pure scCO_2_, under stretching forces of 0, 3, 6, 9, 12, and 15 N, with the same conditions of sample 2 in Table 1. At least 60 samples were tested for all specimens. The vertical bars represent the standard deviations.

In the supercritical environment, the mobility of the fiber molecular segments increased. When there was no stretching, the regularity of the molecular chain of the aramid fibers was destroyed. Thus, the crystallinity of the aramid fibers decreased, and the interplanar spacing also increased, which resulted in a decrease in tensile strength. To solve the problem, the aramid fibers were stretched prior to pure scCO_2_ treatment under different stretching forces of 0, 3, 6, 9, 12 and 15 N. It can be seen from Figure 5 that when the stretching forces increased, the tensile strength of the aramid fiber first increased and then decreased. Except for that of 15 N, the tensile strength of the aramid fiber was lower than that of the original untreated fiber. In the case of the stretching force of 9 N, the tensile strength increased by 13.8% from 3.144 to 3.577 GPa—the biggest improvement.

**Figure 6:**
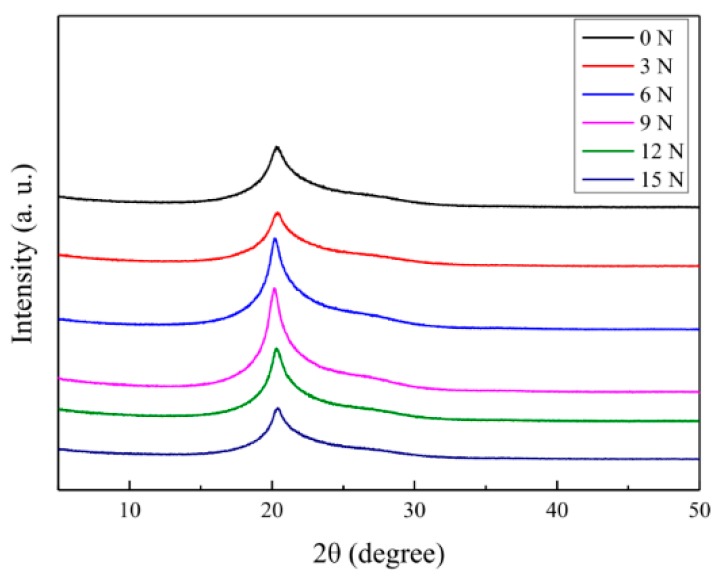
XRD spectra of the aramid fiber under different tensile conditions in pure scCO_2_ under stretching forces of 0, 3, 6, 9, 12 and 15 N, with the same conditions of sample 2 in Table 1.

To investigate the crystallinity characteristic of the aramid fiber after scCO_2_ treatment under stretching, the XRD spectra of different aramid fibers were measured, as displayed in Figure 6. According to the XRD spectra, the crystallinity, interplanar spacing and grain size of the aramid fibers could be calculated, as shown in Table 4. After pure scCO_2_ treatment under different stretching treatments, the crystallinity and the grain size of all the sample groups increased, and the interplanar spacing of all the sample groups decreased. The kinetic ability of the aramid fibers’ molecular chains increased due to supercritical carbon dioxide fluid plasticization, and molecular chains regularly arranged and crystallized better under stretching. Moreover, some of the molecular chains in the amorphous region were rearranged, crystallized, and converted into crystallization regions. Those changes contributed to the increase in crystallinity, the increase in grain size, and the decrease in interplanar spacing, which tensile strength benefited from, as shown in Figure 5.

Furthermore, we discussed the effects of different stretching conditions on fiber crystallization and mechanical properties. When the stretching force was not higher than 9 N, the rearrangement and crystallization of the plasticized molecular chain enhanced with the increase of the axial traction force, which led to the gradual increase of crystallinity and the reduction of interplanar spacing. Thus, the tensile strength of the aramid fiber improved after the scCO_2_ treatment under stretching. However, when the stretching condition was higher than 9 N, the surface molecular chains were quickly rearranged and packed, and only a small amount of CO_2_ molecules penetrated the fiber skin layer. Thus, the fiber surface layer got denser and turned into a barrier that prevented more CO_2_ molecules from getting inside the fiber. The limitation of the improvement of the molecular chain’s crystallization resulted in a relative decrease of tensile strength. Besides that, under the condition of 15 N, the tensile strength of the fiber decreased to 3.138 GPa, which may have been caused by the tensile condition being too severe, thus causing the fiber to be damaged to some degree.

### 3.3. Effect of 1,4-Dichlorobutane in scCO_2_ on Mechanical Properties of Aramid Fibers and Aramid Fiber Enhanced Epoxy Resin Composites

**Figure 7:**
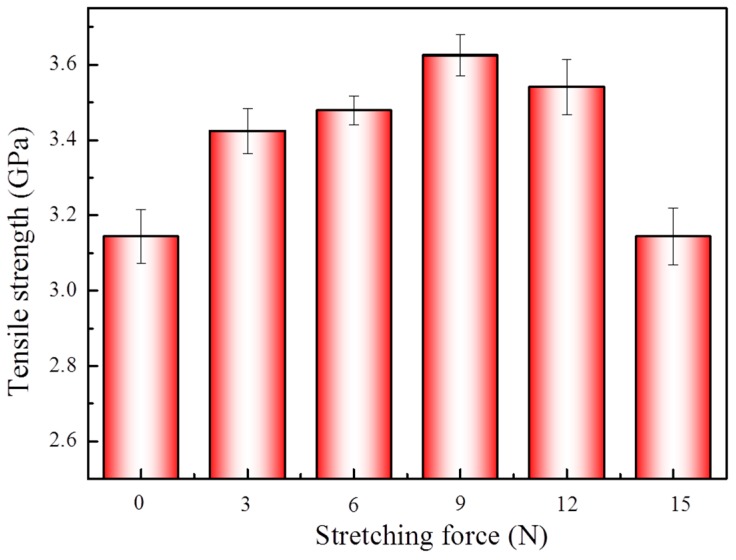
Tensile strength of the fiber treated with the 1,4-dichlorobutane in scCO_2_, under stretching forces of 0, 3, 6, 9, 12 and 15 N, with the same conditions of sample 2 in Table 1. At least 60 samples were tested of all specimens. The vertical bars represent the standard deviations.

The 1,4-dichlorobutane was introduced into the supercritical treatment system under the same stretching conditions of 0, 3, 6, 9, 12 and 15 N, and the tensile strength of the modified aramid fibers of each group are shown in Figure 7. When comparing with the data of the same stretching conditions shown in Figure 5, it can be seen that the introduction of 1,4-dichlorobutane resulted in a further increase in the tensile strength of the fiber. Among them, the highest tensile strength was 3.625 GPa, which was improved by 15.3% from the untreated fibers. The use of 1,4-dichlorobutane as a cross-linking agent could increase the cross-linking degree of molecular chains of the aramid fibers, and the cross-linking reaction was favorable for the tensile strength of the aramid fibers [22,23].

**Figure 8:**
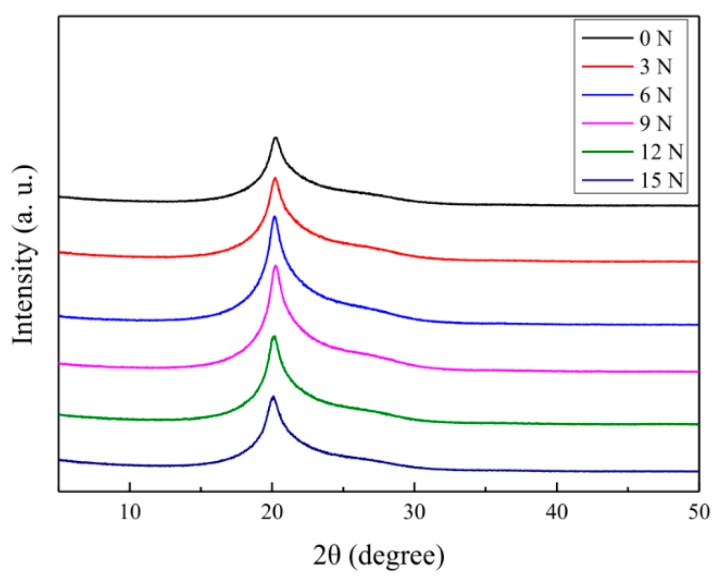
XRD spectra of the aramid fiber after treated with 1,4-dichlorobutane in scCO_2_ under stretching forces of 0, 3, 6, 9, 12 and 15 N, with the same conditions of sample 2 in Table 1.

The XRD spectra of different aramid fibers were measured by 1,4-dichlorobutane treatment in scCO_2_, as displayed in Figure 8, and the crystallinity characteristics were also calculated and are displayed in Table 5. When comparing the crystal data of Table 5 with Table 4, it can be seen that the crystallinity of the experimental group with 1,4-dichlorobutane did not significantly change under the same stretching conditions; however, the grain size relatively increased. Therefore, after the modification with 1,4-dichlorobutane in scCO_2_, the tensile strength of the aramid fibers further increased.

**Figure 9:**
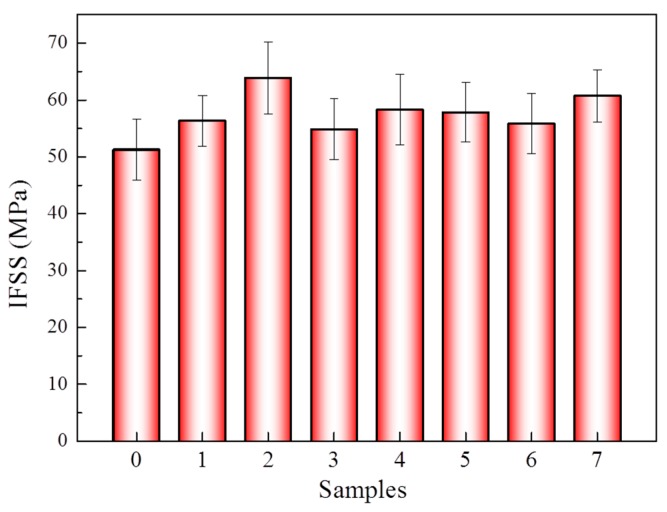
Interfacial shear strength (IFSS) of the aramid/epoxy before and after scCO_2_ treatment with 1,4-dichlorobutane under a static tension of 9 N. (0): untreated fiber; (1–7): modified fibers of samples 1–7 in Table 1. At least 20 samples were tested of all specimens. The vertical bars represent the standard deviations.

The interfacial properties of the aramid fiber-enhanced epoxy resin composites were analyzed after being modified in scCO_2_ with 1,4-dichlorobutane under a static tension of 9 N. The IFSS of all samples increased after the modification comparing with the untreated one, with a maximum of 63.91 MPa, increased by 24.3% from 51.29 MPa of the untreated fibers, as displayed in Figure 9. The significant improvement of the IFSS was due to the rough surface of the aramid fiber after modification, as well as the increased surface energy, as displayed in Figure 2. With the increase of the roughness of the aramid fiber surface, the mechanical locks between the fibers and the epoxy resin were enhanced significantly. Moreover, the higher surface energy was beneficial for the compatibility of the epoxy with the aramid fibers.

## 4. Conclusions

In summary, a novel strategy was developed to improve the mechanical and surface properties of the aramid fiber by treated with 1,4-dichlorobutane in scCO_2_. After modification, the IFSS of aramid the fiber with epoxy resin increased by 24.3% from 51.29 to 63.91 MPa due to its improved surface roughness and surface energy. Furthermore, the tensile strength of the aramid fiber increased by 15.3% from 3.14 to 3.62 GPa, which was attributed to the crystallization improvement of the aramid fiber under stretching forces. This work is of great significance for the simultaneous development of surface modification and mechanical property improvement methods for aramid fibers. In the next step, it is valuable to investigate the comprehensive mechanical properties of aramid fiber-reinforced composites after modification.

## Figures and Tables

**Table 1:** Conditions of supercritical carbon dioxide (scCO_2_) treatment for aramid fiber modification.

Samples	Pressure (MPa)	Time (min)	Temperature (°C)
1	9.0	90	40
2	9.0	90	60
3	9.0	90	80
4	7.5	90	60
5	10.0	90	60
6	9.0	40	60
7	9.0	60	60

**Table 2:** C1s analysis of untreated aramid fibers and sample 2 in Table 1.

Classification	C–C	C–N, C=N	C=O
Untreated fibers	60.37%	29.35%	10.28%
Modified fibers	72.43%	18.90%	8.67%
Ion etched fibers	64.72%	26.45%	8.83%

**Table 3:** C/O atomic ratio and element composition of the Cl of the untreated aramid fibers and sample 2 in Table 1.

Classification	C/O (%)	Cl (%)
Untreated fibers	4.80	0.04
Modified fibers	28.81	1.93
Ion etched fibers	25.27	1.45

**Table 4:** Crystallinity characteristics of the fibers treated under tension in pure scCO_2_.

Stretching Force (N)	Crystallinity (%)	Interplanar Spacing (nm)	Grain Size (nm)
0	80.71	4.394	6.622
3	80.93	4.383	6.758
6	81.43	4.357	6.846
9	84.52	4.346	6.971
12	83.68	4.356	6.912
15	83.07	4.362	6.882

**Table 5:** Crystallinity characteristics of the fibers treated under tension in pure scCO_2_.

Stretching Force (N)	Crystallinity (%)	Interplanar Spacing (nm)	Grain Size (nm)
0	80.71	4.394	6.622
3	80.87	4.372	6.788
6	82.01	4.355	6.899
9	84.49	4.344	7.012
12	83.60	4.349	6.935
15	82.97	4.365	6.901

## Supplementary Materials

The following are available online at https://www.mdpi.com/1996-1944/12/22/3766/s1, Figure S1: SEM images of aramid fiber surface morphology before and after treated with pure scCO_2_. (a) Untreated fiber; (b) modified fiber with pure scCO_2_ under the same condition of sample 2 in Table 1, the scale bar was 2 μm. Figure S2: C1s spectrum and concentrations of correlative functional groups of modified fibers of sample 2 in Table 1 after ion etch for 60 s.
