# Supplementary material for: Improving the Mechanical and Surface Properties of Aramid Fiber by Grafting with 1,4-Dichlorobutane under Supercritical Carbon Dioxide
Source: Materials (Basel). 2019 Nov 16;12(22):3766. doi: 10.3390/ma12223766 (PMC6888609; doi:10.3390/ma12223766)
Supplement: Supplementary file 1 [file materials-12-03766-s001.pdf]

materials

Supplementary Materials

Improving the Mechanical and Surface Properties of Aramid Fiber by Grafting with 1,4-Dichlorobutane under Supercritical Carbon Dioxide

Chuyuan Jia, Chengce Yuan, Zhenyu Ma, Yunzhe Du, Li Liu * and Yudong Huang

MIIT Key Laboratory of Critical Materials Technology for New Energy Conversion and Storage, School of Chemistry and Chemical Engineering, Harbin Institute of Technology, Harbin 150001, China; lurenjia-cn@163.com (C.J.); 16b925019@stu.hit.edu.cn (C.Y.); Mazhenyuhit@outlook.com (Z.M.); duyunzhehit@outlook.com (Y.D.); ydhuang.hit1@aliyun.com (Y.H.)

* Correspondence: liuli@hit.edu.cn; Tel.: +86-0451-86414806

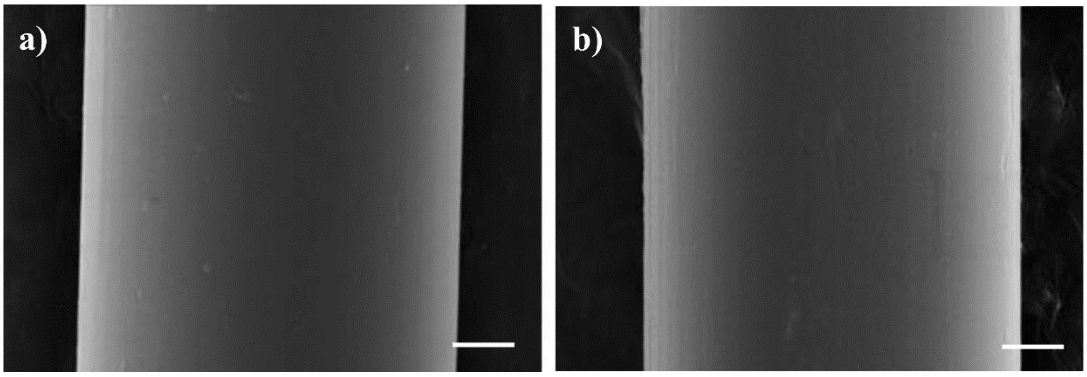

Figure S1. SEM images of aramid fiber surface morphology before and after treated with pure $scCO_2$. (**a**) Untreated fiber; (**b**) modified fiber with pure $scCO_2$ under the same condition of sample 2 in Table 1, the scale bar was 2 μm.

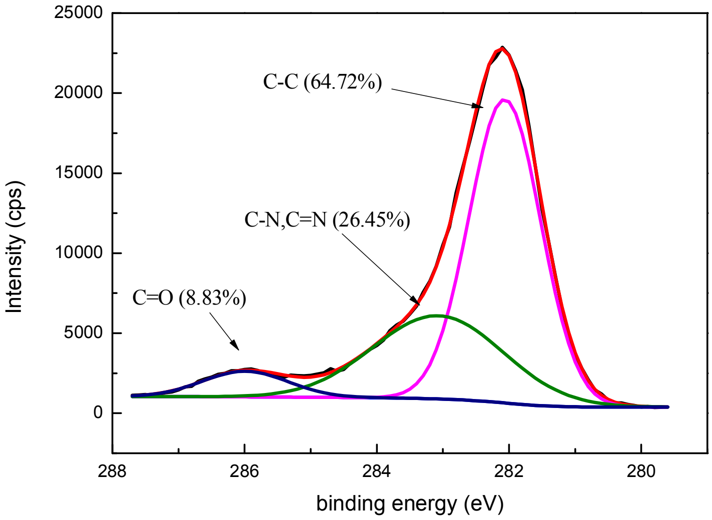

Figure S2. C1s spectrum and concentrations of correlative functional groups of modified fibers of sample 2 in Table 1 after ion etch for 60 s.
